# Methods Used to Evaluate mHealth Applications for Cardiovascular Disease: A Quasi-Systematic Scoping Review

**DOI:** 10.3390/ijerph182312315

**Published:** 2021-11-23

**Authors:** Felix Holl, Jennifer Kircher, Walter J. Swoboda, Johannes Schobel

**Affiliations:** 1 DigiHealth Institute, Neu-Ulm University of Applied Sciences, 89231 Neu-Ulm, Germany; Jennifer.Kircher@student.hnu.de (J.K.); walter.swoboda@hnu.de (W.J.S.); johannes.schobel@hnu.de (J.S.); 2 Institute for Medical Information Processing, Biometry, and Epidemiology, Ludwig Maximilian University of Munich, 81377 Munich, Germany

**Keywords:** mobile health, cardiovascular diseases, evaluation methods

## Abstract

In the face of demographic change and constantly increasing health care costs, health care system decision-makers face ever greater challenges. Mobile health applications (mHealth apps) have the potential to combat this trend. However, in order to integrate mHealth apps into care structures, an evaluation of such apps is needed. In this paper, we focus on the criteria and methods of evaluating mHealth apps for cardiovascular disease and the implications for developing a widely applicable evaluation framework for mHealth interventions. Our aim is to derive substantiated patterns and starting points for future research by conducting a quasi-systematic scoping review of relevant peer-reviewed literature published in English or German between 2000 and 2021. We screened 4066 articles and identified *n* = 38 studies that met our inclusion criteria. The results of the data derived from these studies show that usability, motivation, and user experience were evaluated primarily using standardized questionnaires. Usage protocols and clinical outcomes were assessed primarily via laboratory diagnostics and quality-of-life questionnaires, and cost effectiveness was tested primarily based on economic measures. Based on these findings, we propose important considerations and elements for the development of a common evaluation framework for professional mHealth apps, including study designs, data collection tools, and perspectives.

## 1. Introduction

In 2019, over 331,000 deaths in Germany were attributed to cardiovascular disease (CVD) [1], the treatment of which generates higher medical costs to the German healthcare system than any other single illness, estimated at € 46.4 billion in 2015 [2]. Similarly, in the US, CVD is among the most expensive and most frequent causes of death among the population [3]. Kvedar et al. [4] pointed out the urgent need to develop, optimize, and evaluate programs and technologies that ensure more effective care for patients, where mobile health (mHealth) concepts are likely to play a significant role [5]. The World Health Organization defines mHealth as “Medical and public health practice supported by mobile devices, such as mobile phones, patient monitoring devices, personal digital assistants (PDAs), and other wireless devices” [6].

The 2019 German Digital Healthcare Act (DVG) permitted mobile health applications (mHealth apps)that meet specific requirements to be included the list of reimbursable digital health applications (DiGA list) [7]. Germany is one of the first countries to introduce a standardized mechanism for reimbursing digital health services and its healthcare and medical insurance policy-makers are still working through several challenges. For example, the DiGA list only includes mHealth apps classified as medical devices as defined in the Medical Devices Act administered by the German Federal Institute for Drugs and Medical Devices (BfArM) [8]. While other professional mHealth apps, such as medication reminders or prevention apps, demonstrate both medical benefit and positive care effects, they remain ineligible for reimbursement.

Beyond narrowly defined medical devices, the data and treatment results provided by other professional mHealth apps require equally stringent assessment to ensure reliably high-quality care. Notably, there is currently no established and broadly applicable framework for evaluating mHealth interventions [9].

As a step toward filling this gap, this study examines the criteria and methods for evaluating mHealth interventions for cardiovascular disease discussed in the published literature as a basis for developing a more broadly applicable framework.

## 2. Materials and Methods

In this study, we conducted a quasi-systematic scoping review of methods and criteria used to evaluate cardiovascular disease mHealth apps in the published literature. In a preliminary scoping review, we identified gaps in the literature and synthesized key concepts in a narrative review [10]. Then, in an iterative process, we scoped the literature with refined search terms, performing a final quasi-systematic search with fixed search terms [11].

### 2.1. Preliminary Scoping Review

We conducted a preliminary scoping review of articles of mHealth apps for CVD through an unstructured and open search to generate an overview of existing methods of evaluating mHealth apps for CVD [12] and to confirm the validity of our research objective. The results of this review informed the development of our final search strategy and analysis.

### 2.2. Inclusion and Exclusion Criteria

Our preliminary scoping review revealed various apps designed to reduce the users’ risk of developing cardiovascular disease. These apps focus mainly on reduction and control of risk factors for CVD, such as diabetes, hypertension, chronic obstructive pulmonary disease, nutrition, and physical activity. Based on these results, we derived inclusion and exclusion criteria for the subsequent quasi-systematic scoping review of publications in German and English evaluating mHealth apps designed for adult patients diagnosed with acquired cardiovascular disease. Table A1 in the Appendix A provides a complete overview of our inclusion and exclusion criteria.

### 2.3. Search Strategy

Our final search followed a quasi-systematic approach. We searched the “PubMed”, “Livivo”, and “ProQuest” databases to identify relevant literature published between 2000 and the beginning of April 2021. The last search took place on 6 April 2021. Using keywords and index terms relevant to cardiovascular disease, mHealth, and evaluation, we developed search strings, which we adjusted for each database. Table A2 in the Appendix A provides a list of our search terms.

### 2.4. Literature Selection

**Figure 1:**
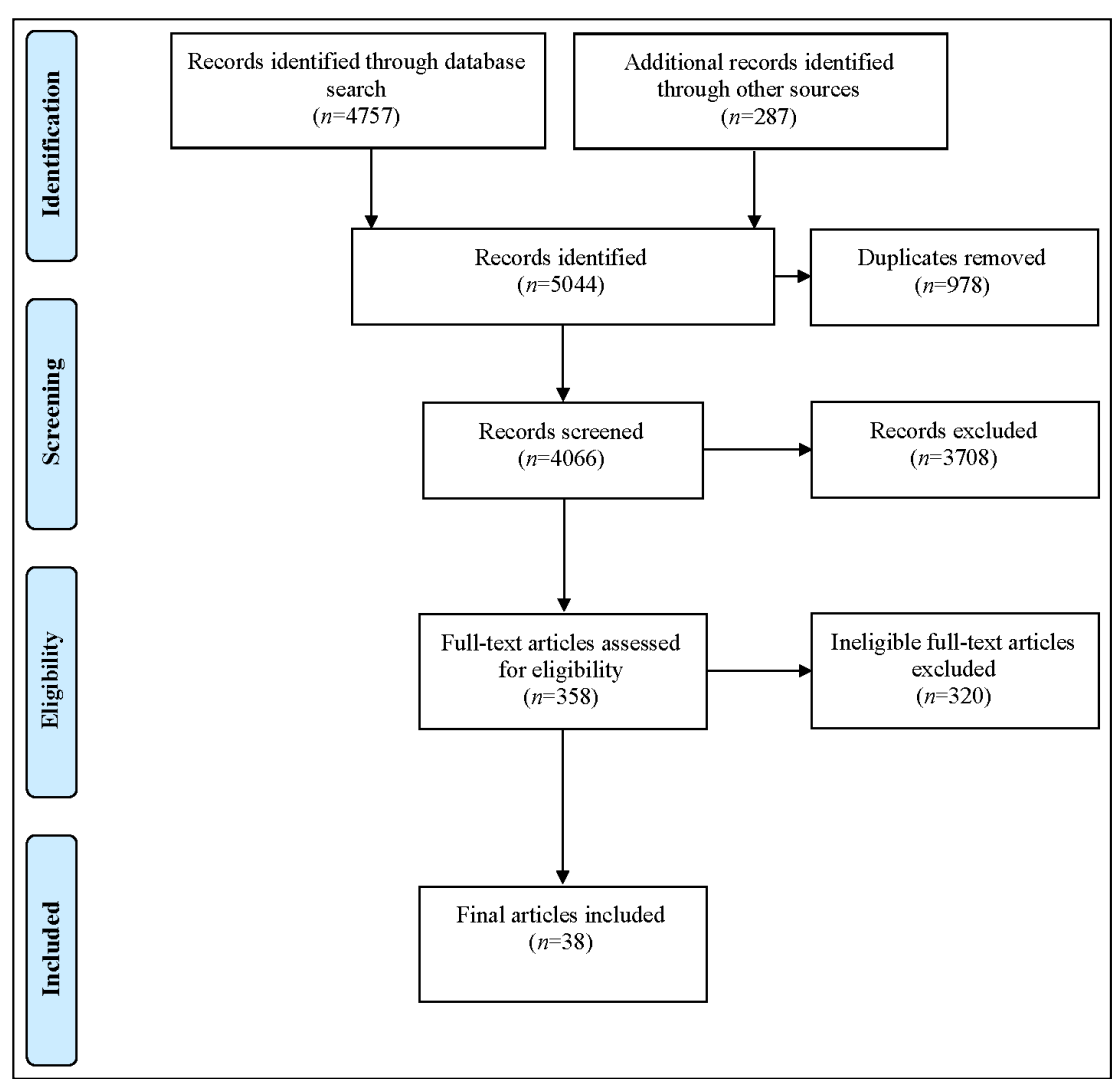
PRISMA flow diagram of the study.

In selecting suitable literature, we applied the Preferred Reporting Items for Systematic Reviews and Meta-Analyses (PRISMA) scheme [13]. The process steps and the results of the study selection are illustrated in Figure 1 below.

After importing our 5044 records into *Covidence*, we excluded 978 duplicates. Then, two scholars independently screened the titles and abstracts of the remaining 4066 entries to identify adherence to previously defined inclusion and exclusion criteria. After resolving inconsistencies by consensus, 3708 studies were excluded. We then undertook a full-text review of the remaining 358 articles, excluding an additional 320 studies because they failed to meet our inclusion criteria. Many of the articles we excluded were study protocols, focused on apps designed only to prevent risk factors, such as high blood pressure or diabetes apps, or assessed apps that rely on implanted sensor technology. Our final sample of *n* = 38 articles was included in the scoping review and approved for data extraction.

### 2.5. Data Extraction and Analysis

In a next step, we extracted data from the studies according to variables, in order to sort and map the literature to reveal patterns, key information, and research gaps in a data chart for subsequent evaluation. The data extraction sheet was developed by two authors based on the findings of the preliminary scoping review and adapted as part of the iterative process to ensure all relevant information from the studies were captured and included in the analysis. To identify evaluation approaches and criteria, we classified the studies into three categories. Interventions carried out using only an app are classified as “mHealth app”; interventions using an app plus at least one additional device, such as an electrocardiogram or smartwatch, are classified as “mHealth system”; and interventions using only text messages are classified as “mHealth text messaging”. Table A3 in the Appendix A summarizes the extracted information as a data chart.

## 3. Results

### 3.1. Characteristics of the Identified Studies

**Figure 2:**
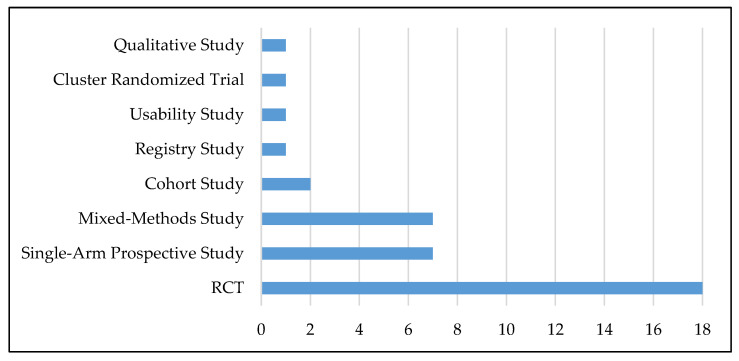
Study designs of the studies identified.

All articles included in our study were published between 2012 and 2020, even though our search spanned 2000 to April 2021. One-third of the articles were published by scholars in the US (*n* = 13), 13% by scholars in Australia, and 10% by scholars in China. Studies with quantitative and qualitative research designs were included in our review. The largest proportion (*n* = 18) consists of randomized controlled trials (RCTs), followed by single-arm prospective studies and mixed-methods studies (each *n* = 7). Figure 2 illustrates the frequency of study designs.

Four of the studies [14,15,16,17] lasted over 12 months, while the shortest study lasted 2 weeks [18]. The largest study had 767 participants [18], while the smallest study had 8 participants [19]. Just over half (57.9%) of the studies reported a retention rate (RR) (the percentage of study participants who remained in the study until the defined end of the study process) of between 90% and 100%, while only four studies [20,21,22,23] reported an RR of below 50%. For analysis purposes, we also tracked the corresponding loss to follow-up (LTFU) (the percentage of study participants who drop out of a study before the defined end of the study process) figure for each study. 

Just over half (52.6%) of the studies focused on mHealth systems (app plus device). The context includes applications for telemonitoring (*n* = 12) as well as for cardiac rehabilitation (CR) (*n* = 8). Seven studies in the mHealth apps (app only) category focused on self-management applications and five focused on CR. In contrast, the smallest share (15.7%) of studies focused on text messaging for self-management purposes (mHealth text messaging category).

### 3.2. Methods and Measurements for Evaluating mHealth Technologies

The studies followed qualitative, quantitative, and mixed-methods designs and the great majority (*n* = 31) analyzed data collected through standardized questionnaires. In most cases (*n* = 33), the overall aim of the research was to assess participants’ perceptions of treatment and subjective health. In addition to general questionnaires on quality of life (e.g., “EQ-5D” [15], “health-related quality of life” [15], illness (e.g., “Self-Care of Heart Failure Index” [24]) or the psychological well-being of the patients (e.g., “8-item Morisky Medication Adherence Scale” [25,26], “Hospital Anxiety and Depression Scale” [20]), specific question sets for digital applications were also used. The Mobile Application Rating Scale (MARS) was frequently applied in assessing mHealth apps [27]. The “Perceived Health Web Site Usability Questionnaire” (PHWSUQ) [28] specifically addresses assessing the usability of websites among elderly participants [29]. Each questionnaire appeared once in the analysis [18,28]. In addition to standardized question sets, self-defined questionnaires (*n* = 3), interviews (*n* = 5), and open-feedback rounds (*n* = 7) were conducted to determine perceptions.

A large proportion of the publications (63%) evaluated mHealth interventions using medical measurements (e.g., blood pressure, pulse, weight), comparing health parameters before and after the intervention. The results were often compared directly between the standard of care and the mHealth intervention (*n* = 15). The medical outcomes were used to assess, among others, the feasibility of the intervention (*n* = 16) and physical activity (*n* = 21). The measurements were either documented by the participants using the mHealth device or determined by healthcare providers using monitoring data or laboratory diagnostics. 

Interactions with the mHealth app on the part of patients (*n* = 19) and health care providers (*n* = 2) were often recorded in usage protocols (*n* = 19) used to draw conclusions about participants’ motivation (*n* = 17), adherence (*n* = 18), and self-efficacy (*n* = 14). In mHealth apps for CR, usage data and logging activities related to login-ins, training, or learning modules were analyzed [30,31]. In one study of an mHealth system for medication adherence [32], the number of times two electronic pill bottles were opened was documented using timestamps.

The usability of mHealth interventions (*n* = 14) was evaluated using several measurement methods and instruments, such as the PHWSUQ and the “System Usability Scale” [33]. A theoretical basis was used in two studies [34,35] to develop the intervention and measure usability. One study adapted the Unified Theory of Acceptance and Use of Technology 2 (UTAUT2) to measure various factors influencing mHealth intervention technology use behavior [36]. In another study [34], the practice of mHealth was prompted by the responsible intervention team as part of a usability test.

Over one-third of the studies (*n* = 14) investigated the effectiveness and efficiency of mHealth for new clinical treatments. Several studies relied on various key performance indicators (KPIs) in assessing mHealth effectiveness (*n* = 11), including, most frequently, hospital readmission, length of hospital stay, number of doctor visits, and hospital admittance due to heart defects. Less attention was paid to mortality and personnel resources required for monitoring. Two studies [37,38] undertook cost-effectiveness analyses. A small number of studies used application-specific indicators, such as data management [38,39], communication between users [38,40], app features [18,41], design characteristics [42], or technology and algorithm analyses [43].

## 4. Discussion

The integration of mHealth apps into healthcare structures is a relatively young field of investigation: the analysis shows that the oldest two studies [14,24] date back less than 10 years, probably due to relatively recent and rapid developments in mobile technologies. The relevance of the research topic of mHealth systems and their evaluation is supported by the large number of publications that we found, and a large body of research exists for health applications for certain manageable illnesses and conditions, such as diabetes, high blood pressure, and obesity-related health problems. Most of the studies included in the analysis were randomized controlled trials, thus providing high-quality evidence-based results and high proof of efficacy [44]. 

### 4.1. Patient Empowerment in mHealth Interventions for CR

Overall, our results show that mHealth interventions for cardiac rehabilitation (CR) can be used to reduce or manage coronary heart disease (CHD) and potentially contribute to secondary prevention by empowering heart attack survivors to monitor their risk factors themselves and act accordingly. We find that by using self-management functions, patients can participate actively in their care process and take more responsibility for their health [45]. We thus identify self-efficacy and motivation as key indicators for evaluating mHealth interventions and in an evaluation framework. This recommendation underscores Schwab et al.’s discussion of approaches to developing mHealth applications and the importance they attribute to increasing awareness and empowerment among patients and healthcare professionals [46].

### 4.2. Usage Behavior and Motivation

Our results show that the retention rate and LTFU are suitable measures of motivation and commitment among mHealth intervention users. The fact that more than half of the studies identified had a very high retention rate indicates an overall positive perception of mHealth interventions among users. Our results indicate that usage protocols provide reliable insights into usability, acceptance, and user motivation levels. We also identify the benefits of adapting the Unified Theory of Acceptance and Use of Technology 2” (UTAUT2) to fit the mHealth application use context: the modified construct includes seven factors influencing intention to use a telemonitoring system, together with the independent variables age, gender, and experience influencing the factors. 

### 4.3. Quantitative and Qualitative Research Methods

While both quantitative and qualitative research methods can be used to collect data, almost all included studies use standardized validated questionnaires and scales, enabling the analysis and comparison of large samples and yielding comparable quantifiable results. Using validated tools is cost and time efficient [47]. Since quantitative research methods often allow little room to interpret the questions, the research framework should include open questions, such as semi-structured interviews or focus groups [48]. Our results illustrate the benefits of combing quantitative and qualitative research methods, particularly in assessing patient satisfaction with the intervention.

### 4.4. Quality Assessment

The Mobile Application Rating Scale (MARS) [18] has been used as an instrument to assess the quality of mHealth apps according to the following quality indicators: engagement, functionality, aesthetics, information quality, and subjective app quality [27]. Terhorst et al. [49] demonstrated the suitability and validity of these indicators and recommended using the instrument to increase transparency for stakeholders and patients. While an mHealth intervention evaluation framework should include app quality criteria, the quality assessment should not be limited to subjective user feedback but rather should include data quality and interoperability with other devices and interfaces.

### 4.5. Privacy and Data Security

Data security and privacy are important to patients and legally protected. Schnall et al. [50] found a decrease in trust in mHealth solutions and data transfer over time and Zhou et al. [51] showed that some patients refuse to use mHealth applications because of security concerns, loss of interest, or hidden costs. Despite these concerns, our results show that little attention has been paid to data management, such as data transfer between health care providers and participants, data privacy, and data security. An mHealth app evaluation framework should assess the app’s data protection systems carefully and communicate the results transparently. 

### 4.6. Economic Evaluation

Performance measures, such as hospital readmissions, are an important indicator of the effectiveness and efficiency of mHealth systems and should be included in an evaluation framework as well. In the CR mHealth intervention context, our results show that mHealth apps can reduce heart failure-related hospital days and studies conducting cost-effectiveness analysis underscore that shortening out- and inpatient stays also cuts healthcare costs [52]. Similarly, Maddison et al.’s [37] post-hoc economic evaluation assessed the costs of implementing and delivering the intervention to calculate the incremental cost-effectiveness ratio (ICER) between costs and quality-adjusted life years (QALYs) gained and to compare the health benefit gains of switching from standard in- and outpatient care to mHealth-supported care. The authors found that mHealth interventions are more cost-effective compared to the standard care and can improve health-related quality of life in an ongoing program. Martín et al. applied a “Hidden Markov Model” to measure cost-effectiveness. Long-term costs and outcomes associated with an illness and a particular health intervention can be estimated over multiple cycles, based on resource use and health outcomes [53]. Martín et al.’s [38] study modeled the different disease states of patients during the mHealth intervention, using economic parameters for the outcome analysis and aligning participants’ health-specific and follow-up data with healthcare costs published by the health care system. Their cost-effectiveness analysis model showed that introducing an mHealth app lowered the overall cost of disease management by 33% of the total cost of disease management [38]. Pavlović et al.’s [54] results are equally striking: introducing mHealth apps can reduce the total expenses related to data collection in medical scenarios by 50%.

## 5. Conclusions

Our scoping review of scholarly articles including criteria and methods of evaluating mHealth apps for cardiovascular disease makes recommendations for developing an evaluation framework for mHealth interventions. In keeping with recent research on the health benefits of active patient involvement in their treatment process, we recommend adopting a user perspective. While various methods and criteria have been used, we recommend quantitative methods using validated standardized questionnaires to generate comparable quantifiable results with a reasonable effort in terms of time commitment and cost. In addition to considering the overall effects of mHealth apps on mental and physical health, we recommend that mHealth intervention evaluations apply usage protocols to understand the patients’ interaction with the application and assess their motivation, engagement, and acceptance of integrating the interventions into healthcare processes sustainably. We also recommend including the retention rate and LTFUs, and adapting use and acceptance constructs, such as UTAUT2, into the mHealth technology use setting by modifying its assessment dimensions accordingly.

In terms of the overall scope and considerations for the development of an mHealth app evaluation framework, we recommend focusing on the added value of an mHealth intervention. Specifically, we recommend laboratory diagnostics and physical tests to assess objective physical health, standardized surveys and semi-structured interviews to assess subjective quality of life, and economic performance and efficiency KPIs, such as hospital readmission data and incremental cost-effectiveness ratios between costs and quality-adjusted life years. Heterogeneity of results by using different standardized surveys and questionnaires could be a major challenge for the analysis and comparisons of the results from such a framework. Therefore, the selection of data collection tools needs to be made carefully.

mHealth app providers, patients, healthcare providers, healthcare systems, and society at large will benefit by applying these recommendations when developing a holistic framework to evaluate mHealth apps and interventions to ensure that they are effective, efficient, empowering, accurate, sustainable, and safe. Such a framework will enable an informed decision when choosing an mHealth app.

## Data Availability

All relevant data can be found in the Appendix A.

## Appendix A

**Table A1:** Inclusion and exclusion criteria based on the PCC elements.

PCC Elements	Inclusion Criteria	Exclusion Criteria
Population	Patients (>18 years) with a diagnosed CHD No limitation of the number of participants, origin, gender of the study participants	Patients who are at risk of coronary heart disease Relatives of cardiovascular patients, e.g., children Comorbid heart disease (e.g., congenital heart defect, heart transplant) Healthy, voluntary study participants
Concept	*mHealth Application*	
	Wearable mHealth applications for patients with CHD Studies using qualitative or quantitative methods to evaluate mHealth applications (e.g., standardized questionnaires, quality guidelines, device data sets, usage logs) No limitation of the evaluation parameters Fully developed mHealth applications	mHealth applications for the use of exclusively: Risk factors (e.g., high blood pressure) Diabetes Chronic Obstructive Pulmonary Disease Pregnancy Nutrition assessment (e.g., food tracking) Sport and Wellness Sensor technology (e.g., implanted sensor) Applications that are only designed for health care providers, e.g., Clinical Assessment Tool A risk screening tool of CHD for the population Pure descriptions of the apps (e.g., system, technical, program, algorithm description)
	*Study Design*	
	Single study designs for evaluating a mHealth intervention for patients with CHD Written in English or German	Study protocols Preliminary studies (e.g., for the development of the app) Reviews (e.g., systematic reviews, scoping reviews) Case studies
Context	No limitation of cultural parameters (e.g., geographical location, social origin, gender-specific interests)	Unpublished literature
	No restriction of the setting, e.g., acute care, primary care, rehabilitation facilities	
	Full texts	

**Table A2:** Search strings and number of results.

Database	Search String	Search Date	Results (*n*)
PubMed	Heart Disease* OR Cardiovascular Disease* AND “Mobile Health” OR “mHealth” OR Smartphone App* AND Evaluation	5 January 2021	2916
Livivo	Cardiovascular disease AND mHealth OR mobile health app AND evaluation	13 January 2021	485
Proquest	(mHealth OR “mobile health” app) AND Evaluation AND cardiovascular disease	13 January 2021	1356
**Total records**			**4757**
+ Additional studies from reference lists of 37 systematic reviews			
Pubmed		6 April 2021	287
**Total records generated by search**			**5044**

**Table A3:** Extracted data of the 38 studies included in the analysis.

Country [Ref]	Setting	Type of Intervention	Study Design	Type(s) of Evaluation	Evaluation Indicators	Evaluation Methods
Canada [24]	Home-based and hospital	mHealth system devices: mobile phone, weight scale, blood pressure monitor, ECG recordings	RCT Sample size *n* = 100 Duration: 6 months Retention rate: 94% Loss to follow-up: 6	Feasibility Medical Outcomes Comparison with standard of care Utilization Clinical Management Quality of Life Effectiveness/Efficiency	Clinical endpoints Physical well-being Health parameters (BP, weight, ECG) Hospital KPIs application: Patient perception /feedback Clinicians’ interaction	Medical measurements Standardized questionnaires Collection of hospital KPI data
USA [34]	Home-based	mHealth app	Usability study Sample: *n* = 15 Duration: - Retention rate: 87% Loss to follow-up: 2	Acceptability Usability Medical outcome Self-efficacy	Clinical endpoints: Physical activity Application: Task completion success Mobile technology use Patients’ interaction	Interviews Standardized questionnaires Open feedback Usability testing Guidance by UTAUT2 construct
USA [30]	Home-based and cardiac rehabilitation	mHealth system devices: app, monitoring dashboard	Single-arm prospective study Sample: *n* = 18 Duration: 3 months Retention rate: 72% Loss to follow-up: 5	Feasibility Engagement Acceptability Medical outcome	Clinical endpoints: Health parameters (BP, functional capacity, safety) Application: Patients’ interaction with app Patient perception/feedback	Open feedback Usage logs
Belgium [31]	Home-based and cardiac rehabilitation	mHealth app	Mixed-methods study Sample: *n* = 32 Duration: 4 months Retention rate: 88% Loss to follow-up: 4	Comparison of usual care Engagement Effectiveness Usefulness Medical outcome Quality of life	Clinical endpoints: Physical activity Health parameters Application: Patients’ perception/feedback Patients’ interaction	Interviews Standardized questionnaires Medical measurements Usage logs
China [39]	Home-based	mHealth app	Cluster randomized trial Sample: *n* = 209 Duration: 3 months Retention rate: 80% Loss to follow-up: 42	Usability Feasibility Acceptability Medical outcome Safety accuracy/consistency Quality of life Self-efficacy	Clinical endpoints: Health parameters Psychological well-being Application: Patients’ perception/feedback Knowledge Data management	Open Feedback Medical measurements Standardized questionnaires Questionnaires (self-defined) Collection of cointervention data (medical outcome data)
USA [55]	Home-based and hospital	mHealth system devices: wireless ECG, app	Cohort study Sample: *n* = 46 Duration: 6 months Retention rate: 76% Loss to follow-up: 11	Comparison of usual care Feasibility Quality of life Medical outcome Self-efficacy	Clinical endpoints: Physical and psychological well-being Health parameters (ECG) Application: Patient perception/feedback	Standardized questionnaires Medical measurements Usability testing
USA [40]	Home-based and hospital	mHealth system devices: tablet, Bluetooth-weight scale, pulse wave blood pressure wrist monitor	Mixed-methods study Sample: *n* = 28 Duration: 3 months Retention rate: 89% Loss to follow-up: 3	Feasibility Comparison of usual care Usability Acceptability Medical outcome Clinical management Self-efficacy	Clinical endpoints: Health parameters Physical well-being Physical activity Application: Adherence Patients’ perception/feedback Clinicians’ interaction	Standardized questionnaires Medical measurements Interviews
USA [41]	Home-based	mHealth app	RCT Sample: *n* = 60 Duration: one month Retention rate: 92% Loss to follow-up: 5	Comparison of telehealth Medication adherence Feasibility Quality of life Acceptability Self-efficacy	Clinical endpoints: psychological and physical well-being Application: App features Patients’ interaction	Questionnaires (self-defined) Usage logs
New Zealand [56]	Home-based	mHealth system devices: mobile phone, device for internet support	RCT Sample: *n* = 171 Duration: 6 months Retention rate: 92% Loss to follow-up: 14	Medical outcome Self-efficacy	Clinical endpoints: Physical well-being Physical activity (leisure-time and walking) Health parameters	Standardized questionnaires Medical measurements
USA [42]	Home-based	mHealth app	Mixed-methods study Sample: *n* = 12 Duration: one month Retention rate: 92% Loss to follow-up: 1	Feasibility Usability Quality of life Self-efficacy Acceptability Effectiveness/efficacy Medical outcome	Clinical endpoints: Health parameters Hospital KPIs Application: Patient perception/feedback Message characteristics	Open feedback Medical measurements Standardized questionnaires Collection of hospital KPI data
Australia [35]	Home-based	mHealth system devices: app, tracking tools (accelerometer, wrist-worn Fitbit Flex), web-based program	Cohort Study Sample: *n* = 21 Duration: 4 months Retention rate: 62% Loss to follow-up: 8	Feasibility Usability Medical outcome Self-efficacy Quality of life Medical outcome	Clinical endpoints: Health parameters Physical activity Psychological well-being Application: Mobile Technology Use Patient perception/Feedback Resource Requirements Patients’ interaction	Medical measurements Standardized questionnaires Usage logs
USA [16]	Home-based	mHealth—Text messaging	RCT Sample: *n* = 84 Duration: 12 months Retention rate: 99% Loss to follow-up: 1	Comparison of usual care Medication adherence	Clinical endpoints: Physical well- Physical activity Application: Patients’ interaction	Usage logs Medical measurements Questionnaire
USA [57]	Home-based and hospital	mHealth system devices: apps, bp cuff, scale, dashboard, medicine software platform	Registry study Sample: *n* = 60 Duration: one month Retention rate: 97% Loss to follow-up: 2	Feasibility Acceptability Effectiveness/efficacy Medical outcome	Clinical endpoints: Health parameters Hospital KPIs Application: Patients’ interaction	Collection of hospital KPI data Usage logs
Australia [23]	Home-based	mHealth app	RCT Sample: *n* = 166 Duration: 3 months Retention rate: 92% Loss to follow-up: 14	Medication adherence Feasibility Comparison of usual care Adherence Acceptability Medical outcome	Clinical endpoints: Health parameters Application: Patient perception/feedback	Standardized questionnaires Open feedback Medical measurements
Malaysia [25]	Home-based	mHealth -text messaging	RCT Sample: *n* = 62 Duration: 2 months Retention rate: 97% Loss to follow-up: 2	Medication adherence Medical outcome Effectiveness/efficacy	Clinical endpoints: Health parameters Hospital KPIs Application: Patient perception/feedback	Medical measurements Standardized Questionnaires Collection of Hospital KPIs data
USA [32]	Home-based	mHealth system devices: mobile phone, electronic pillbox, web-based platform	RCT Sample: *n* = 90 Duration: one month Retention rate: 93% Loss to follow-up: 6	Medication adherence Feasibility Acceptability Comparison of usual care Usability	Application: Patient perception/feedback Patients’ interaction	Standardized Questionnaires Usage logs
USA [58]	Home-based and hospital	mHealth system devices: tablet, blood pressure cuff, weight scale, web-based platform	Single-arm prospective study Sample: *n* = 21 Duration: 3.2 months Retention rate: 95% Loss to follow-up: 1	Engagement Effectiveness/efficacy Acceptability Feasibility Usability (incl. ease of use) Quality of life Medical outcome	Clinical endpoints: Health parameters Hospital KPIs Application: Patient perception/feedback Patients’ interaction	Questionnaires (self-defined) Medical measurements Usage logs Collection of hospital KPIs data Standardized questionnaires
Norway [33]	Home-based and cardiac rehabilitation	mHealth app	Single-arm prospective study Sample: *n* = 14 Duration: 3 months Retention rate: 100% Loss to follow-up: 0	Feasibility Quality of life Usability Effectiveness/efficacy	Clinical endpoints: Physical well-being Hospital KPIs Application: Patient perception/feedback Patient satisfaction Adherence Patients’ interaction	Standardized questionnaires Open feedback Usage logs Collection of hospital KPIs data
New Zealand [37]	Home-based	mHealth System Devices: mobile phone, web-based platform	RCT Sample: *n* = 171 Duration: 6 months Retention rate: 89% Loss to follow-up: 18	Comparison of usual care Effectiveness Self-efficacy Engagement Medical outcome Quality of life Economic outcome	Clinical endpoints: Physical activity Health parameters Application: Patient perception/feedback Cost and Cost-effectiveness	Medical measurements Standardized questionnaires Economic measurements
Norway [15]	Home-based and cardiac rehabilitation	mHealth app	RCT Sample: *n* = 113 Duration: 12 months Retention rate: 98% Loss to follow-up: 2	Comparison of usual care Medical outcome Quality of life	Clinical endpoints: Health parameters Application: Patient perception/feedback Patient satisfaction	Medical measurements Standardized questionnaires
France [59]	Home-based	mHealth—text messaging	RCT Sample: *n* = 521 Duration: one month Retention rate: 96% Loss to follow-up: 22	Medication adherence Comparison of usual care	Clinical endpoints: Health parameters Application: Patient perception/feedback	Open feedback Medical measurements
China [28]	Home-based and hospital	mHealth system devices: apps, smart tracking devices (bp cuff, weight scale, wearable ECG), remote monitoring service platform	Single-arm prospective study Sample: *n* = 70 Duration: 4 months Retention rate: 94% Loss to follow-up: 4	Usability Medical outcome Satisfaction Engagement Feasibility	Clinical endpoints: Physical activity Health parameters Application: Mobile Technology Use Patient perception/feedback Health care provider experience Relatives’ experience Patients’ interaction	Interviews Standardized questionnaires Usage logs Medical record entries Medical measurements
Netherlands [60]	Home-based and hospital	mHealth system devices: app, weight scale, blood pressure monitor, rhythm monitor, step counter	RCT Sample: *n* = 200 Duration: - Retention rate: 90% Loss to follow-up: 20	Medical outcome Feasibility Satisfaction Effectiveness/efficacy Comparison of usual care	Clinical endpoints: Health parameters Hospital KPIs Application: Patients’ interaction Patient perception/feedback	Medical measurements Standardized questionnaires Collection of hospital KPIs data Medical record entries Usage logs
Canada [61]	Home-based and hospital	mHealth system devices: app, weight scales, blood pressure monitors	Single-arm prospective study Sample: *n* = 315 Duration: 6 months Retention rate: 90% Loss to follow-up: 30	Quality of life Effectiveness/efficacy Medical outcome Self-care	Clinical endpoints: Hospital KPIs Health parameters Application: Patient perception/feedback	Collection of hospital KPIs data Standardized questionnaires Medical measurements
USA [21]	Home-based and cardiac rehabilitation	mHealth app	Qualitative Study Sample: *n* = 16 Duration: 2.2 months Retention rate: 25% Loss to follow-up: 12	Feasibility Acceptability Medical outcome Medication adherence Engagement Effectiveness/efficacy	Clinical endpoints: Health parameters Physical activity Hospital KPIs Application: Patients’ interaction Patient perception/feedback	Medical measurement Usage logs Collection of hospital KPIs data
China [19]	Home-based	mHealth—text messaging	RCT Sample: *n* = 767 Duration: 6.4 months Retention rate: 95% Loss to follow-up: 37	Effectiveness/Efficacy Quality of life Self-efficacy Medication adherence	Clinical endpoints: Hospital KPIs Health parameters Application: Patient perception/feedback	Collection of hospital KPIs data Standardized questionnaires
USA [62]	Home-based	mHealth system	RCT Sample: *n* = 90 Duration: one month Retention rate: 93% Loss to follow-up: 6	Medication adherence Self-efficacy	Clinical endpoints: Psychological well-being Application: Patients’ interaction Patient perception/feedback	Standardized questionnaires Usage logs
Spain [38]	Home-based	mHealth app	RCT Sample: *n* = 630 Duration: - Retention rate: 86% Loss to follow-up: 86	Economic outcome Engagement Quality of life Efficacy	Application: Cost-effectiveness Patient satisfaction Data management Communication	Economic measurements
Australia [18]	Home-based	mHealth app	Mixed-methods study Sample: *n* = 8 Duration: between 2 and 4 weeks Retention rate: 75% Loss to follow-up: 2	Usability	Clinical endpoints: Physical activity Application: Patient perception/feedback App features Mobile technology use	Standardized questionnaires Interviews
Canada [17]	Home-based and hospital	mHealth system devices: app, weight scales, blood pressure monitors	Mixed-methods study Sample: *n* = 231 Duration: 12 months Retention rate: 87% Loss to follow-up: 30	Usability Adherence Engagement Medical outcome	Clinical endpoints: Health parameters Application: Mobile technology use Adherence Patients’ interaction Patient perception/Feedback	Guidance by UTAUT2 construct interviews Usage logs Standardized questionnaire Medical measurements
China [63]	Home-based and hospital	mHealth—text messaging	Mixed-methods study Sample: *n* = 190 Duration: 3 months Retention rate: 93% Loss to follow-up: 13	Feasibility Usability Acceptability Medication adherence Economic outcome	Clinical endpoints: Physical activity Application: Patient satisfaction Patient perception/feedback costs	Standardized questionnaires Open feedback Economic measurements
Israel [64]	Home-based and cardiac rehabilitation	mHealth system devices: mobile phone, smartwatch, monitoring system	Single-arm prospective study Sample: *n* = 22 Duration: 6 months Retention rate: 100% Loss to follow-up: 0	Feasibility Safety Adherence Effectiveness/efficacy Medical outcome Usability	Clinical endpoints: Physical activity Hospital KPIs Health parameters Application: Patient satisfaction Patients’ interaction Patient perception/Feedback	Collection of hospital KPIs data Medical measurements Usage logs Standardized questionnaires
Norway [20]	Home-based	mHealth system devices: mobile phone, web-based platform	RCT Sample: *n* = 69 Duration: 3 months Retention rate: 28% Loss to follow-up: 50	Comparison of usual care Usability Self-efficacy Adherence	Clinical endpoints: Physical activity Psychological well-being Application: Patients’ interaction Patient perception/Feedback	Standardized questionnaires Usage logs
Australia [43]	Home-based and cardiac rehabilitation	mHealth system devices: app, blood pressure monitor, weight scale, web-based platform	RCT Sample: *n* = 66 Duration: 6 months Retention rate: 77% Loss to follow-up: 15	Medical outcome Feasibility Security	Clinical endpoints: Physical activity Health parameters Psychological well-being Application: Technology and algorithm	Medical measurement Standardized questionnaires
New Zealand [65]	Home-based and cardiac rehabilitation	mHealth system devices: mobile phone, web-based platform, pedometer	RCT Sample: *n* = 123 Duration: 6 months Retention rate: 94% Loss to follow-up: 7	Comparison of usual care Medical outcome Medication adherence Self-efficacy Acceptancy	Clinical endpoints: Physical activity Psychological well-being Health parameters Application: Patient perception/feedback	Standardized questionnaire Open feedback Guidance following on the mHealth development and evaluation framework
Australia [23]	Home-based	mHealth App	Mixed-methods study Sample: *n* = 58 Duration: 3 months Retention rate: 26% Loss to follow-up: 43	Comparison of usual care Medication adherence Acceptability Utilization Engagement	Clinical endpoints: Health parameters Application: Patient perception/feedback Patients’ interaction	Standardized questionnaire Usage logs Open feedback
Spain [14]	Home-based and cardiac rehabilitation	mHealth system devices: mobile phone, web-based platform, sphygmomanometer, glucose, and lipid meter	RCT Sample: *n* = 203 Duration: 12 months Retention rate: 90% Loss to follow-up: 21	Usefulness Medical outcome Quality of life	Clinical endpoints: Health parameters Psychological well-being Application: Patient perception/feedback	Medical measurement Standardized questionnaires
USA [22]	Home-based	mHealth—text messaging	Single-arm prospective study Sample: *n* = 15 Duration: one month Retention rate: 40% Loss to follow-up: 9	Feasibility Acceptability Medication adherence Adherence Engagement	Application: Patient perception/feedback Patient satisfaction Patients’ interaction	Usage logs Standardized questionnaires
